# Seroprevalence of Toscana Virus in Blood Donors, France, 2007

**DOI:** 10.3201/eid1705.101052

**Published:** 2011-05

**Authors:** Nadège Brisbarre, Houssam Attoui, Pierre Gallian, Paola Di Bonito, Colomba Giorgi, Jean-Francois Cantaloube, Philippe Biagini, Mhammed Touinssi, Francois Jordier, Philippe de Micco

**Affiliations:** Author affiliations: Université de la Méditerranée, Marseille, France (N. Brisbarre, P. Gallian, J.-F. Cantaloube, P. Biagini, M. Touinssi, F. Jordier, P. de Micco);; Institute for Animal Health, Woking, UK (H. Attoui);; Instituto Superiore di Sanità, Rome, Italy (P. Di Bonito, C. Giorgi)

**Keywords:** Viruses, Toscana virus, blood donors, France, sandflies, vector-borne infections, letter

**To the Editor:** Toscana virus (TOSV) is an arthropod-borne RNA virus (family *Bunyaviridae* and genus *Phlebovirus*) transmitted by sandflies in Mediterranean countries. TOSV causes acute meningitis and meningoencephalitis in patients. In France, cases of TOSV infections involving resident populations and cases imported by tourists traveling in TOSV-endemic countries have been reported (*1**,**2*); the virus has also been isolated from local wild-caught sandflies (*1*). The fact that TOSV has been isolated from human blood on several occasions (*2*) suggests a potential risk exists for transmitting the virus through blood transfusion or organ transplantation. We investigated the presence of TOSV antibodies in a sample of the healthy population, blood donors from southeastern France.

We tested plasma collected from 729 blood donors in 7 French territorial divisions during the summer of 2007. Plasma donors were analyzed according to their address of residence in each territorial division. Information related to these donors is reported in the Table.

**Table:** Prevalence of antibodies against Toscana virus in blood donors, France, 2007*

Demographic characteristic	No. donors	% IgG-positive samples	% IgM-positive samples
Age, y			
<30	211	11.8	3.8
30–39	133	11.3	3
40–49	156	11.5	2.6
50–60	158	10.8	4.4
>60	71	12.7	1.4
Sex			
F	353	13.9	2.5
M	376	9.3	4
French territorial division†			
Alpes de Haute Provence	29	10.3	3.4
Hautes Alpes	64	6.25	1.6
Alpes Maritime	111	19	0
Bouches du Rhône	143	12.6	2.8
Corsica	115	8.7	8.7
Var	154	8.4	1.9
Vaucluse	113	13.3	4.4
Total	729	11.5	3.3

*Plasma samples were determined as positive by using an ELISA to detect immunoglobulin (Ig) G against Toscana virus (absorbance cutoff value [optical density at 450 nm (OD450)]>0.47) and IgM (absorbance cutoff value of OD450>0.15). Mean age of seropositive blood donors were the following: women, 40 y (SD 13.73 y); men, 41.8 y (SD 13.71 y). †French territorial division elevations: Alpes de Haute Provence, 280–3,412 m; Hautes Alpes, 430–4,101 m; Alpes Maritimes, 0–3,143 m; Bouches du Rhône, –2–1,042 m; Corsica, 0–2,706 m; Var, 0–1,714 m; Vaucluse, 12–1,909 m.

Presence of immunoglobulin (Ig) G and IgM against TOSV was investigated by using a commercial enzyme immunoassay kit (EIA Enzywell Toscana virus IgG and IgM; DIESSE Diagnostica Senese S.p.A*.,* Siena, Italy) developed by using the recombinant nucleocapsid (N) protein of TOSV. This serologic test was validated in a previous study that revealed high specificity and sensitivity (*3*).

Our results showed that 84 (11.5%) of 729 plasma samples were positive for IgG against TOSV N protein. Twenty-four (3.3%) plasma samples were positive for IgM, and 5 (0.7%) were positive for IgG and IgM (Table).

To confirm the ELISA results, IgG-positive samples were further subjected to Western blot (WB) analysis by using TOSV (isolate H/IMTSSA [*2*])–infected cell lysate (*4*). In 233 (32%) of samples, we detected a protein of molecular mass compatible with that of the N protein. A previously reported antibody-positive control was used to validate the WB assay (*5*). Our WB analysis showed a reduced sensitivity when compared with results of ELISA. After chemical/heat treatment of the protein samples, WB will only detect the linear epitopes on the N protein, while ELISA detects both linear and conformational epitopes. Furthermore, a less recent exposure of the blood donor population to the virus would have resulted in weaker N protein detection by WB as a consequence of a lower antibody titer. However, we cannot exclude some aspecific cross-reactivity as a consequence of well-conserved N protein sequence among the genus.

Finally, to detect TOSV RNA, we processed IgM-positive plasma samples by reverse transcription–PCR (*6*). The finding of IgM is an indication of a recent exposure to the virus and hence a possible presence in blood. Our PCR did not detect any viral RNA in the samples. Such negative results could indicate either cleared viremia or a low viral load, below the sensitivity limit of the test.

Serologic information obtained in our study confirms the circulation of TOSV in southeastern France. Factors such as commercial exchange and movement of humans, animals, and arthropods between France and Italy may explain the highest prevalence observed (18.8%) in the Alpes Maritimes territorial district, which borders Italy. Our results regarding this area appear of the same order of magnitude as those reported in the general Italian population (>20%) (*1*).

Geographic and climatic conditions (e.g., temperature, humidity), factors that affect vector distribution and abundance (*7*), could explain the lower prevalence found in the mountainous districts (collectively ≈400–2,000 meters in elevation). The lower temperatures in these districts may also affect the ability of vectors to efficiently transmit the virus in the field (*8*).

TOSV prevalence in Corsica, an island in the Mediterranean Sea, was unexpectedly high. In this region, ≈8.7% (10 donors of 115) of the population sampled showed an IgG- or IgM-positive response. In the other districts, the IgM seroprevalence did not exceed 4.4%. The vector that transmits TOSV is known to be present in this area (*7*), and TOSV infections have been reported on nearby Sardinia (*9*). The elevated IgM titer in the population in Corsica could indicate 1) recent virus contacts; 2) recent infections with a new TOSV strain circulating in Corsica; or 3) presence of related phleboviruses that are inducing cross-reactivity in the N protein–based IgM ELISA.

Our results demonstrate that 14.1% (IgG and IgM) of the healthy population (blood donors) in France living on the Mediterranean border have been in contact with TOSV and show asymptomatic or mild, unidentified symptoms, as it is the case for many other arbovirus infections (*10*). Such findings raise concerns about the risks of virus transmission to virus-naive persons by blood transfusions and organ transplants.

Further investigation is needed to better assess how widespread TOSV is in populations. For example, a donor–recipient investigation might confirm virus transmission by blood transfusion, and studies related to the behavior of sandfly vectors, virus biology, and mammalian reservoir hosts could help define populations at higher risk for exposure.
